# Cost-optimal nZEB reform strategies and the influence of building orientation for Mediterranean university buildings: case study of the University of Málaga

**DOI:** 10.1016/j.heliyon.2022.e09020

**Published:** 2022-03-03

**Authors:** Daniela Carolina Da Costa Duarte, Carlos Rosa-Jiménez

**Affiliations:** Universidad de Málaga - Escuela Técnica Superior de Arquitectura de Málaga. Campus El Ejido, Plaza El Ejido, nº 2, Málaga, Andalucía, 29013, Spain

**Keywords:** Nearly zero-energy building (nZEB), Energy simulation, Cost-optimal level, Energy savings, Retrofitting strategies

## Abstract

The Energy Performance of Buildings Directive (EPBD) requires the transformation of European buildings into nearly zero-energy buildings (nZEBs) before 2050 as a mitigation strategy against the imminent climate emergency. This paper aims to determine the cost-optimal nZEB reform strategy for Spanish university buildings located in the Mediterranean climate, evaluating the influence of the building's orientation on nZEB cost optimality. The present work carries out a case study on three similar buildings at the University of Málaga. The optimal cost-efficiency nZEB reform strategy is determined, rigorously maintaining the formal design of the building through energy simulations with Sefaira Systems software under the EPBD framework. Six reform options were proposed to determine the cost-optimal strategy meeting nZEB requirements for each building. The results show that the most profitable strategy is to improve the efficiency of the thermal envelope and the HVAC system, reducing the energy consumption of the studied buildings by 85–93%. Although a general strategy for nZEB renovations was identified, building orientation proved to be a governing factor in energy performance. As such, the cost-optimal reform strategy was found to be specific to each particular building.

## 1 Introduction

An imminent climate emergency is a prominent international challenge that requires transformative action towards greenhouse gas (GHG) emission reductions (Masson-Delmotte et al., 2018). The European Union (EU) and its Member States (MS) have committed to a minimum 40% GHG emissions reduction target before 2030 while increasing energy efficiency by at least 32.5% and renewable energy penetration by at least 32% compared to reference 1990 levels (European Commission, 2014). Buildings account for approximately 40% of the final energy consumption and 36% of the CO_2_ emissions in the EU (Eurostat, 2019). At a national level, 30% of the electricity consumption in Spain is attributed to the building sector, 41% of which corresponds to non-residential buildings (Government of Spain, 2020). As such, the energy rehabilitation of the building sector presents a great opportunity for achieving EU climate change mitigation goals.

Under this context, the Energy Performance of Buildings Directive (EPDB) Recast establishes nearly zero-energy buildings (nZEBs) as the new EU construction standard after 2021. It requires that the existing public building stock be transformed to nZEB at an average annual rate of 3%, considering a regional-specific cost-optimal retrofit level (European Parliament, 2010). The effectiveness and profitability of nZEB renovation strategies depend on the type of building, location, and climate (D'Agostino and Parker, 2018).

The rehabilitation of academic buildings could provide a great impetus towards meeting nZEB targets, as tertiary buildings represent 25% of the existing building stock in the EU, 17% of which is intended for educational use (Economidou et al., 2011). Public university buildings in Spain are of particular interest, as their primary non-renewable energy consumption is approximately 2–16 times greater than the nZEB standard (Medrano et al., 2018). Vested interest has been placed in the Mediterranean climate zone, as it covers more than 20% of the territorial surface of Spain (Cárcel-Carrasco et al., 2017), and most southern European countries are not adequately prepared to implement the nZEB standard (López-Ochoa et al., 2019).

Although numerous studies have been published in the last five years on energy rehabilitation strategies for academic buildings situated in temperate EU countries (Table 1), no previous research has been conducted on nZEB reform strategies for university buildings located in southern Spain. Furthermore, no previous research has yet to be conducted on the influence of Spanish academic building orientation on nZEB cost optimality. Under this context, the present work aims to determine the cost-optimal nZEB reform strategy for Spanish university buildings located in the Mediterranean climate, evaluating the influence of the building's orientation on nZEB cost optimality by investigating the following three questions:

1. What is the optimal energy efficiency for the thermal envelope of university buildings in the Mediterranean climate?
2. What is the cost-optimal renovation strategy required to transform existing Mediterranean university buildings to nZEB without altering the architectural form?
3. What is the influence of building orientation on the cost-optimal nZEB renovation strategy for existing university buildings in the Mediterranean climate?

**Table 1:** Published studies on nZEB rehabilitation of academic buildings in temperate EU countries.

Study	Country	Location	KCCS	Typology
ZEMedS (2015a)	France	Montpellier	Csa	School
ZEMedS (2015b)	France	Bédarieux	Cfa	School
ZEMedS (2015c, 2015d)	Greece	Athens	Csa	School
Congedo et al. (2016)	Italy	Puglia region	Csa	School
ZEMedS (2015e)	Italy	Colle di Val d’Elsa	Csa	School
ZEMedS (2015f)	Italy	San Miniato Basso	Csa	School
Rospi et al. (2017)	Italy	Matera	Csa	School
Dalla Mora et al. (2017)	Italy	North East Region	Cfa	School
Salvalai et al. (2017)	Italy	Lecco	Cfa	School
Zinzi et al. (2016)	Italy	Cesena	Cfa	School
Berardi et al. (2017)	Spain	Barcelona	Csa	School
Gaitani et al. (2015)	Spain	Catalonia	Csa	School
ZEMedS (2015g)	Spain	Badalona	Csa	School
López-Ochoa et al. (2019)	Spain	Logroño	Cfb	School
ZEMedS (2015h)	Spain	Avià	Cfb	School
Soutullo Castro et al. (2019)	Spain	Gijón	Cfb	University
Ascione et al. (2017, 2015)	Italy	Benevento	Csa	University
Ferrari and Beccali (2017)	Italy	Milan	Cfa	University
Semprini et al. (2016)	Italy	Bologna	Cfa	University
**Köppen Climate Classification System (KCCS):**				
Csa = Mediterranean climate with hot summers				
Cfa = Humid subtropical climate				
Cfb = Oceanic climate				

A case study was conducted on three representative lecture halls of the University of Málaga in southern Spain to answer these questions through thermodynamic energy simulations with Sefaira Systems software. These three contemporary buildings feature an almost identical floor plan and occupancy profile, but are each oriented in a distinct direction, presenting a unique opportunity for evaluating the influence of building orientation on cost-optimal nZEB reform strategies.

## 2 Background

Both the Paris Agreement and the EPBD have impulsed the publication of numerous studies on cost-optimal nZEB reform strategies for EU academic buildings, including those conducted by D'Agostino and Parker (2018) throughout Europe and Ascione et al. (2019) in the Mediterranean region. Table 1 enlists previous studies on nZEB renovations in academic buildings located in Mediterranean European countries by academic typology, climate, and location, namely, Spain, Italy, Greece, and France. The closest study was performed by Soto Francés et al. (2020) on nZEB school reformations under the European SHERPA Project. Only two studies (Ascione et al., 2015, 2017) analyse university buildings in the Mediterranean climate (Köppen classification Csa and Csb). However, no studies have been previously conducted on nZEB refurbishments for universities located in southern Spain.

In the Spanish context, studies exist on the cost-optimal nZEB refurbishment methods for residential buildings (Aguacil et al., 2017; Las-Heras-casas et al., 2021), as well as comparative studies on the evolution of the Spanish regulative framework for energy savings pertaining to the residential and academic building sectors (López-Ochoa et al., 2021). Soutullo Castro et al. (2019) developed a methodology based on dynamic simulation tools and a decision matrix for identifying the construction costs and burdens of individual retrofit measures aiming to achieve nZEB status among Spanish schools. Specifically concerning university buildings in Spain, new constructions such as the LUCIA building at the University of Valladolid have not met the nZEB definition established by the EPBD (Rey-Hernández et al., 2018), despite being designated the most efficient building in Europe and the second most efficient building worldwide in 2015 (Davis, 2016).

Table 2 presents benchmark cost-optimal nZEB reform strategies applied throughout different academic buildings in Mediterranean Europe. There is a definite pattern in the identified optimal nZEB retrofit strategies, promoting elevated thermal envelope insulation, energy-efficient equipment, and on-site renewable energy generation. Reduced values of thermal transmittance and solar heat gain coefficients prove to be an indispensable strategy, as all benchmark nZEB reform strategies include the installation of double-glazed, low-emissivity windows. All studies recommend increasing the insulation of facades, with external thermal insulation systems being the most common solution. Not all studies document the need for increasing roofing insulation levels, concluding that the optimal insulation type depends on the roof's structural composition, with external insulation being the preferred method for flat roofs.

**Table 2:** Benchmark nZEB renovation strategies in academic buildings in temperate EU countries.

Reference nZEB refurbishment case study			nZEB renovation strategy																						
			Facade						Windows		Solar protection		Roof				HVAC system					Lighting			Energy
Study	Year built	Floor area (m^2^)	External XPS insulation	External EPS insulation	External natural fibre insulation	Cool coating plaster	Internal plaster insulation	Internal natural fibre insulation	Double pane glazing	Low emissivity glazing	External mobile slats	Interior curtains	External XPS panel insulation	External EPS tile insulation	Cool coating paint	Internal natural fibre insulation	High efficiency electric heat pump	High efficiency biomass heater	High efficiency geothermal heat pump	High efficiency earth-air heat exchanger	Mechanical ventilation with heat recovery	LED lighting	Natural daylighting through skylights	Smart lighting, daylight and occupation sensors	Photovoltaic (PV) plant
Berardi et al. (2017)	1980s	1,641	•	-	-	-	-	-	•	•	-	-	•	-	-	-	-	•	-	-	-	•	-	-	•
Gaitani et al. (2015)	1977	1,366	•	-	-	-	-	-	•	•	•	-	•	-	-	-	-	-	•	-	•	•	-	-	•
Ascione et al. (2017)	1990s	6,459	•	-	-	•	-	-	•	•	•	-	•	-	•	-	•	-	-	-	-	-	-	-	•
Rospi et al. (2017)	1992	3,484	-	-	-	-	-	•	•	•	-	-	-	-	-	•	•	-	-	-	-	-	-	-	-
Congedo et al. (2016)	1977	1,077	-	-	•	-	-	-	•	•	-	-	-	-	-	-	-	-	•	-	•	-	-	-	•
ZEMedS (2015c)	1982	1,832	-	•	-	•	-	-	•	•	-	•	-	•	•	-	-	-	-	-	•	•	-	-	•
ZEMedS (2015g)	1979	1,147	-	•	-	-	-	-	•	•	•	-	-	-	-	•	-	-	-	-	•	•	-	-	•
ZEMedS (2015e)	1975	2,500	-	•	-	•	-	-	•	•	-	•	-	•	•	-	-	-	-	-	-	•	-	-	•
Rey-Hernández et al. (2018)	2014	5,920	-	-	-	-	•	-	-	-	•	-	-	-	-	-	-	•	-	•	•	•	•	•	•

Regarding the optimal reform strategy for heating, ventilation, and air conditioning (HVAC) systems, upgrading existing systems has been deemed necessary only when the current system is of very low efficiency. The optimal type of HVAC system is correlated with the type of renewable energy available on-site. Studies conducted in areas of high geothermal activity recommend geothermal heat pumps, areas with direct access to biomass material promote biomass boilers or combined heat and power systems, and areas of high solar irradiation recommend high-efficiency electric heat pumps. These studies tend to discourage daylighting as a reform strategy, as the construction of new windows and skylights entails a major investment cost that is not sufficiently compensated by long-term lighting electricity savings. On the other hand, LED lighting solutions appear to be complementary - rather than essential - strategy.

Photovoltaic (PV) panel installation has proven fundamental for nZEB academic building renovations in Mediterranean climate zones. Gallo et al. (2014) assert the feasibility of nZEB renovations in Spain due to the recent acquisition cost reductions for this technology, coupled with significant advances in the administrative, technical, and economic conditions for renewable electricity self-consumption in the country.

Previous comparative studies focus on the impact of diverse building typologies and climates on cost-optimal nZEB reform strategies. However, no studies have yet been performed on the influence of building orientation on nZEB reform cost optimality, particularly for university buildings in southern Spain. The present study presents a unique opportunity to fill this research gap. Three of the most important existing lecture halls of the University of Málaga are identical in design and use, but are each oriented in a different direction.

## 3 Methodology

The EPBD defines a nZEB as a high-energy-performance building with minimal operational primary energy consumption that is almost completely compensated by renewable energy generated in situ or nearby over an annual balance period (European Parliament, 2010). Whereas final energy refers to the consumption by the end-user, primary energy refers to the energy generated at its source; that is, the final energy consumed by the building plus transformation, transport, distribution, and storage losses. Final energy consumption is converted to primary energy consumption through the primary energy conversion factor, which has a value of 1 for renewable energy and 1.954 for non-renewable energy in mainland Spain (IDAE, 2016).

However, the EPBD does not define minimum performance standards for nZEB and requires MS to define cost-optimal national or regional thresholds according to the building typology and the climate zone. For tertiary buildings situated in the Mediterranean climate, Recommendation (EU) 2016/1318 suggests a maximum total primary energy consumption rate of 90 kWh/m^2^yr. The non-renewable share should not surpass 30 kWh/m^2^yr (European Commission, 2016). The latest revision of the Spanish Building Code (CTE) came into force in September 2020 and defined the following thresholds for tertiary nZEBs located in the Mediterranean climate: maximum total primary energy consumption rate of 155 + 9CFI kWh/m^2^yr with a maximum non-renewable share of 55 + 8CFI kWh/m^2^yr, whereby CFI corresponds to the average internal energy load per unit area of the building in W/m^2^ (Ministerio de Fomento, 2019).

The current EPBD legislative framework seeks to adapt the nZEB definition to individual MS contexts (Cuniberti, 2017). Still, the resulting variability raises the need to develop a harmonized comparison methodology and a standardized nZEB performance criterion at the EU level (D'Agostino and Parker, 2018). In the absence of a harmonized criterion, this work adopts the energy consumption thresholds proposed in Recommendation (EU) 2016/1318 with the aim of increasing the comparability of results against studies carried out in other MSs of similar climatic conditions, regardless of the average building internal load. Additionally, CTE thresholds are more than double the recommendation (EU) 2016/1318 values, and the most stringent nZEB definition should be adopted in light of the progressively increased level of demand in environmental regulation.

**Figure 1:**
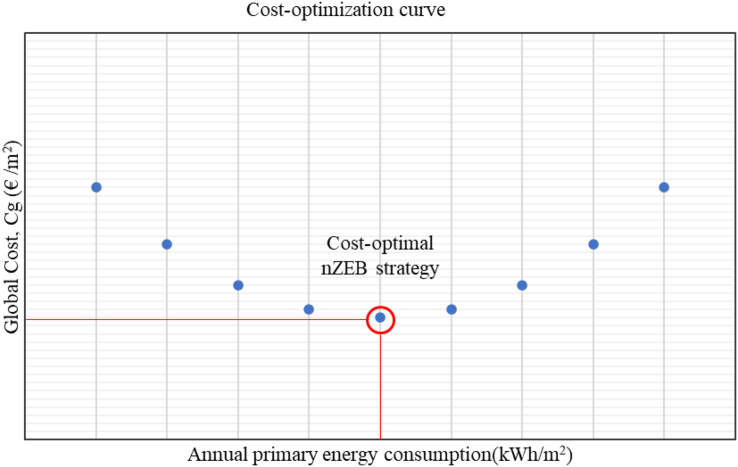
Cost-optimal nZEB renovation curve.

The cost-optimal level for nZEB renovations follows the comparative methodological framework established in Commission Delegated Regulation (EU) No 244/2012, whereby the global cost (Cg) of each renovation strategy considered is graphed as a function of the annual total primary energy consumption of the building. The cost-optimal strategy corresponds to the lowest point of the resulting curve as shown in Figure 1 (European Commission, 2012) (BPIE, 2010, p. 13).

The calculation procedure of the global cost is established in UNE-EN 15459 (AENOR, 2018). It is defined as the total sum of the investment cost of the nZEB renovations plus the annual costs of electricity, operation, maintenance, and replacement. For a macroeconomic analysis, Cg also considers the monetization of annual CO_2_ emissions associated with the non-renewable energy consumption of the building. Cg is expressed as a single total amount per square metre of built surface in net present value (NPV) for the year the energy rehabilitation is carried out. The involved equations and variable definitions are presented in Tables 3 and 4, respectively.

**Table 3:** Equations used to determine the cost-optimal level of nZEB renovations.

#	Name	Description
1	Primary energy	${\text{Q}}_{\text{p,i}}={\text{Q}}_{\text{f,i}}\cdot {\text{f}}_{\text{elct}}$
2	Global cost	${\text{C}}_{\text{g}}\left(\tau_{0}\right)=\frac{{\text{C}}_{\text{i}}+\sum_{\text{j}=1}^{\text{n}}\left[\sum_{\text{j}=1}^{\text{τ}}\left({\text{C}}_{\text{a,i}}\left(\text{j}\right)\cdot {\text{R}}_{\text{d}}\left(\text{i}\right)+{\text{C}}_{\text{c,i}}\left(\text{j}\right)\right)-{\text{V}}_{\text{f,τ}}\cdot {\text{R}}_{\text{d}}\left(\tau \right)\right]}{\text{SU}}$
3	Annual cost	${\text{C}}_{\text{a,i}}\left(\text{j}\right)={\text{C}}_{\text{e,i}}\left(\text{j}\right)+{\text{C}}_{\text{m,i}}\left(\text{j}\right)+{\text{C}}_{\text{f,i}}\left(\text{j}\right)+{\text{C}}_{\text{so,i}}\left(\text{j}\right)$
4	Electricity cost	${\text{C}}_{\text{e,i}}={\text{Q}}_{\text{p\_no\_ren,i}}\cdot {\text{T}}_{\text{i}}$
5	Electricity rate	${\text{T}}_{\text{i}}={\text{T}}_{{\text{τ}}_{\text{0}}}\cdot {\left(1+{\text{RX}}_{\text{e}}/100\right)}^{\text{i}-1}$
6	Discount rate	${\text{R}}_{\text{d}}\left(\text{i}\right)={\left(\frac{1}{1+\left(\text{r}/100\right)}\right)}^{\text{i}}$
7	CO_2_ monetization	$\sum_{\text{i ​}=\text{ ​}1}^{\text{τ}}{\text{C}}_{\text{c,i}}\left(\text{j}\right)=\sum_{\text{i ​}={\text{τ}}_{0}}^{2025}\left({\text{t}}_{{\text{CO}}_{2}\text{eq,i}}\cdot {\text{M}}_{\text{<}2025}\right)+\sum_{\text{i ​}=2026}^{2030}\left({\text{t}}_{{\text{CO}}_{2}\text{eq,i}}\cdot {\text{M}}_{2025-2030}\right)+\sum_{\text{i}=2031}^{\text{τ}}\left({\text{t}}_{{\text{CO}}_{2}\text{eq,i}}\cdot {\text{M}}_{\text{>}2030}\right)$
8	CO_2_ emissions	${\text{t}}_{{\text{CO}}_{2}\text{eq,i}}={\text{Q}}_{\text{f\_no\_ren,i}}\cdot {\text{C}}_{\text{elct}}$

**Table 4:** Definition of variables for the determination of the cost-optimal level of nZEB renovations.

Symbol	Definition	Unit
Q_p,i_	Annual primary energy consumption in year i	kWh
Q_f,i_	Annual final energy consumption in year i	kWh
f_elct_	Primary energy conversion factor	-
C_g_ (τ_0_)	Global cost in NPV for the year of initial investment	€/m^2^
C_i_	Initial inversion cost, excluding incentives	€
C_a,i_(j)	Annual costs for component j in year i	€/year
R_d_(i)	Discount rate for year i	%
C_c,i_(j)	Annual CO_2_ monetization for year i	€
V_f,τ_(i)	Final value of component j at the end of the building lifespan	€
SU	Building built area	m^2^
τ	Building lifespan	years
τ_0_	Starting year of renovation	-
i	Year in question	-
j	nZEB renovation component in question	-
n	Total number of nZEB renovation components	-
C_e_(j)	Annual electricity cost for component j in year i	€/year
C_m_(j)	Annual maintenance cost for component j in year i	€/year
C_f_(j)	Annual operation cost for component j in year i	€/year
C_so_(j)	Annual replacement cost for component j in year i	€/year
Q_p_no_ren,i_	Annual non-renewable primary energy consumption in year i	kWh
T_i_	Electricity rate in year i	€/kWh
T_τ0_	Electricity rate in the initial investment year	€/kWh
RX_e_	Primary energy cost evolution rate	%
r	Inflation rate	%
${\text{t}}_{{\text{CO}}_{2}\text{eq,i}}$	Annual CO_2_ emissions for non-renewable energy consumption in year i	tCO_2_
M < 2025	CO_2_ cost before year 2025	€/tCO_2_
M2025−2030	CO_2_ cost from ear 2025 to 2030	€/tCO_2_
M > 2030	CO_2_ cost after year 2050	€/tCO_2_
Q_f___no_ren__,i_	Annual non-renewable final energy consumption in year i	kWh
C_elct_	CO_2_ conversion factor	-

The research mechanism involved energy simulations with Sefaira Systems software, a simplified online platform for simulating building energy performance that provides comparative results between numerous design options within a few minutes (Li, 2017). Sefaira Systems is a user-friendly interface for the EnergyPlus simulation engine, one of the most robust and widely accepted energy simulation tools in the research community (Corrado and Fabrizio, 2019). An energy model of the current state of the studied buildings was developed following in-person inspections and a thorough blueprint evaluation. The models and current consumption results were validated against historic energy bills of the University of Málaga, with a percent error less than 4%, indicating good reliability of the results.

Six reform options were proposed and optimized individually for each building in terms of cost efficiency following Commission Delegated Regulation (EU) No 244/2012 and UNE-EN 15459 procedures explained above (European Commission, 2012), and (AENOR, 2018), respectively. The optimized options were then combined in a total of 31 reform combinations to determine which combinations meet the nZEB requirements for each building. Cost-optimization was then performed among the combinations meeting the nZEB requirements to identify the cost-optimal strategy for each building. The scope of the energy models conducted under this study encompasses (i) on-site energy production and (ii) energy demands for climatization and lightning. Domestic hot water needs are excluded from the scope of the study, given that the evaluated buildings are academic lecture halls with minimal hot water use.

## 4 Case study

### 4.1 Building description

**Figure 2:**
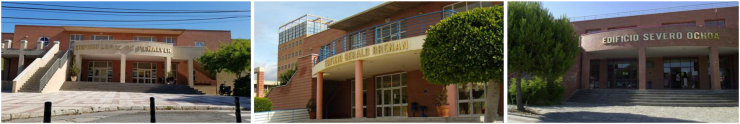
From left to right, Aulario López de Peñalver, Aulario Gerald Brenan, and Aulario Severo Ochoa.

**Figure 3:**
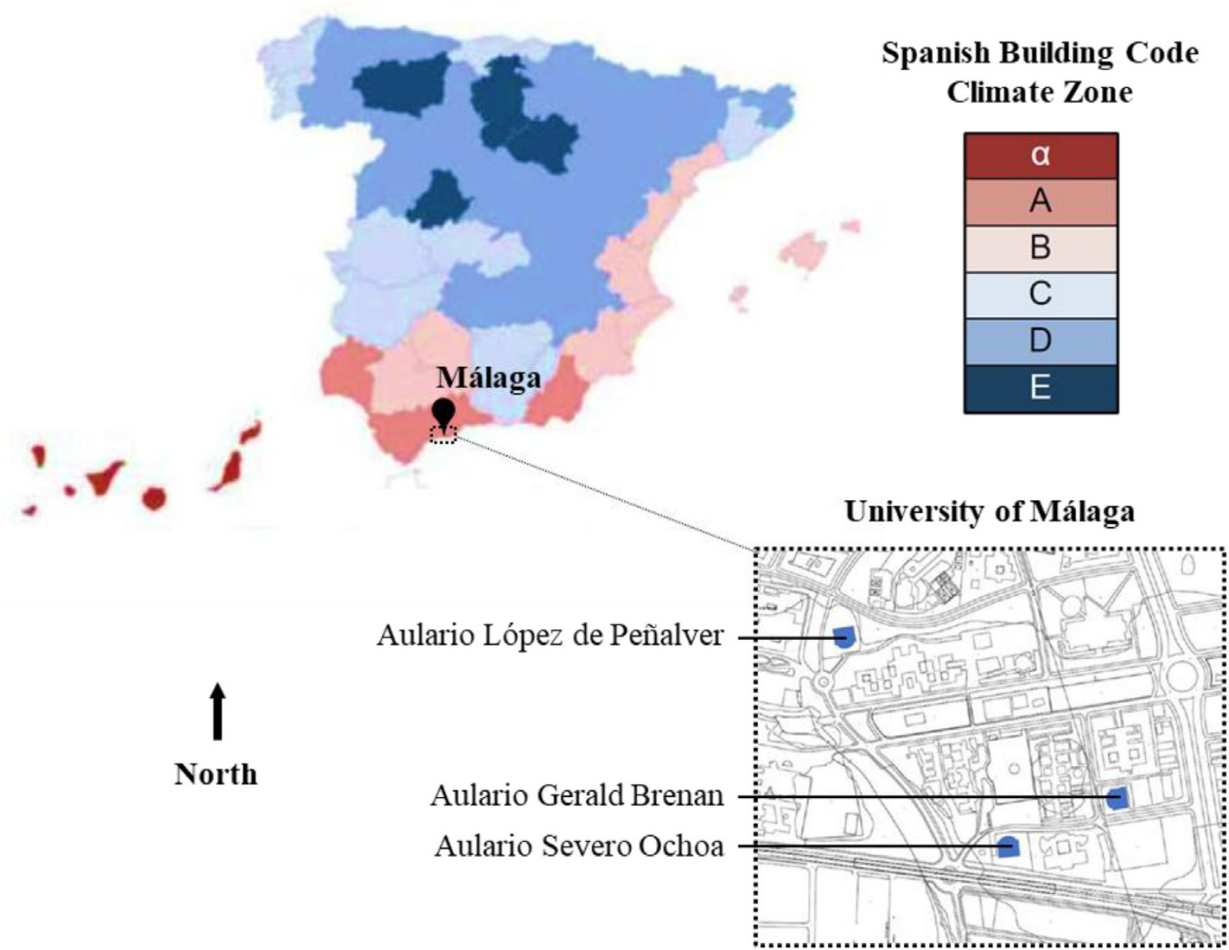
Location of the case study.

This case study evaluates three university lecture halls located in Campus Teatinos at the City of Málaga, Spain, namely, the Aulario Lopez de Peñalver, Aulario Gerald Brenan, and Aulario Severo Ochoa (Figure 2). The City of Málaga is classified as a “Climate Zone A″ according to DB-HE 2019, “Climate Csa - Mediterranean Climate with Hot Summers” according to the Köppen Climate Classification System, and “Mediterranean Climate” according to Commission Recommendation (EU) 2016/1318 (Figure 3).

**Figure 4:**
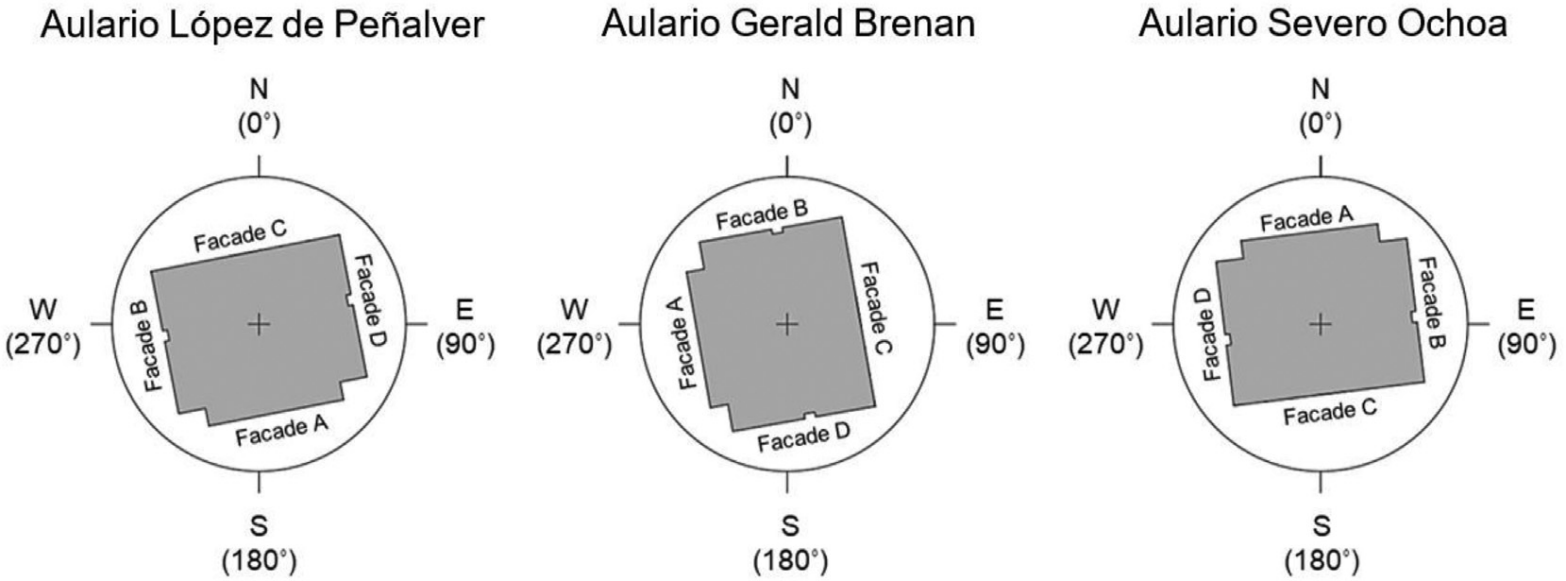
Orientation of the studied buildings. Building entrance through Facade A.

The three buildings were built in 1994 with almost identical floor plans and occupancy profiles. The architectural form consists of a two-storied regular parallelepiped containing a series of classrooms and offices distributed in a U-shape around a central double-height gallery illuminated by a large pyramid-shaped skylight. The Aulario Severo Ochoa also features a semibasement with additional classrooms and a dedicated storage area. Key geometric aspects of each building are presented in Table 5. Each building is oriented differently, enabling the evaluation of building orientation on nZEB retrofit optimality (Figure 4).

**Table 5:** Key geometric parameters of the studied buildings.

Geometric parameter		López de Peñalver	Gerald Brenan	Severo Ochoa
Total floor area (m^2^)		3,195	3,195	4,853
Total envelope area (m^2^)		4,939	4,939	5,682
Building volume (m^3^)		13,586	13,586	20,048
Shape factor		0.36	0.36	0.28
Facade A	Orientation	S (169°)	W (260°)	N (353°)
	Surface (m^2^)	387	387	565
	%Windows	22%	22%	15%
	Shading element	Eave	Eave	None
Facade B	Orientation	W (259°)	N (350°)	E (83°)
	Surface (m^2^)	377	377	550
	%Windows	19%	15%	11%
	Shading element	Exterior blinds	Exterior blinds	Exterior blinds
Facade C	Orientation	N (349°)	E (80°)	S (173°)
	Surface (m^2^)	473	473	692
	%Windows	25%	25%	19%
	Shading element	Exterior blinds	Exterior blinds	Interior curtains
Facade D	Orientation	E (79°)	S (170°)	W (263°)
	Surface (m^2^)	377	377	550
	%Windows	22%	18%	10%
	Shading element	Exterior blinds	Exterior blinds	Interior curtains
Roof	Surface (m^2^)	1,669	1,669	1,669
	%Windows	10%	10%	10%
Floor slab	Surface (m^2^)	1,657	1,657	1,657

The current thermal envelope was built according to the NBE-CT-79 Spanish Building Code in force at the time of construction. The buildings have a non-trafficable inverted flat roof without air chambers and 30 mm extruded polyethylene (XPS) insulation for a total thermal transmittance of 0.84 W/m^2^K. The slab in contact with the ground has a thermal transmittance of 1.69 W/m^2^K with unknown thermal insulation or impermeabilization layers. The facades are composed of double-layered brick walls with nonventilated air chambers and 40 mm of internal fibreglass insulation with a total thermal transmittance of 0.54 W/m^2^K.

**Figure 5:**
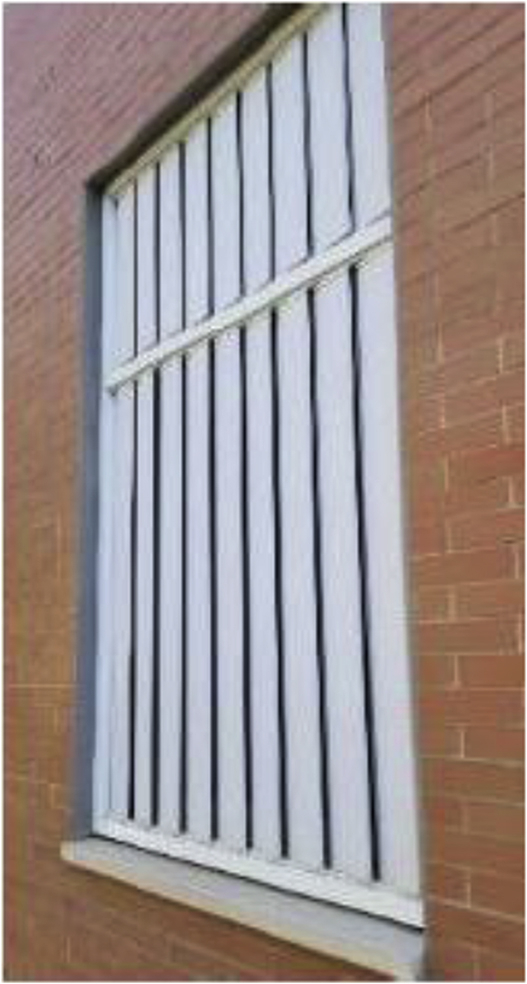
Exterior blinds at Aulario López de Peñalver.

**Figure 6:**
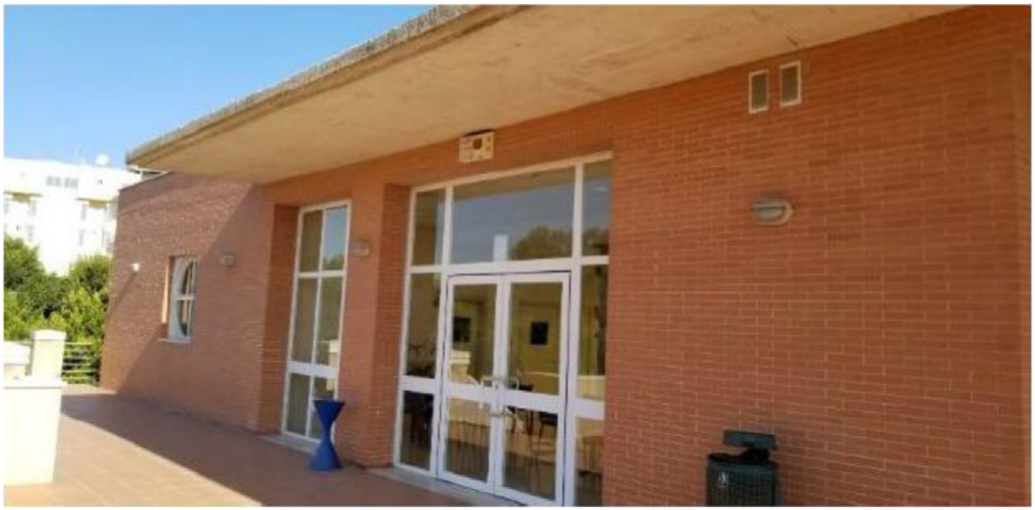
Eave covering the entrance of Aulario López de Peñalver.

Windows are single-paned equipped with metal frames without thermal breaks, and have a global heat gain coefficient of 0.85 and a thermal transmittance of 5.7 W/m^2^K. A combination of shading elements (eaves, exterior blinds, and interior curtains) is currently used to protect the windows for solar incidence and have been incorporated within the Sefaira Systems energy simulation models. The windows of Facades B, C, and D are equipped with manually operated exterior blinds consisting of 145 mm wide vertical PVC louvres (Figure 5). Facade A at both Aularios López de Peñalver and Gerald Brenan feature a reinforced concrete overhang (18 m × 2 m) which protects the glazed doors from the sun in summer, but allows the sun to shine through in winter (Figure 6).

An important source of uncertainty that can significantly influence energy simulation results is the definition of the constructive walls and openings in the energy simulation tool, precisely the building envelope thermal resistance layers necessary to solve the key energy balances and heat transfers for estimating the annual energy consumption. Soutullo Castro et al. (2019) argue that the best option is to specify each element of the building envelope, including the presence of thermal bridges. The influence of possible thermal bridges that may be present at Aularios López de Peñalver, Severo Ochoa, and Gerald Brenan has been excluded from the scope of this study for the following reasons: (1) there is a lack of documentation on specific constructive details of the buildings, for which no information on the possible thermal bridges is available, and (2) Sefaira Systems software does not allow for the modelling of thermal bridges. Per Soutullo Castro et al. (2019), an average U-value was introduced for each facade wall, which increases the uncertainty of the modelling process.

Despite this important source of uncertainty, model validations against historic energy bills show an uncertainty inferior to 4% in current building energy consumption, indicating good reliability of results, given that an acceptable uncertainty range for an accurate energy simulation model lies between ±10% and ±30% (Soutullo Castro et al., 2019).

The HVAC system consists of an electric heat pump with aCOP of 2 and an EER of 1.8. Due to the age of the buildings and a lack of appropriate documentation, no information is available on the SCOP and SEER of the current HVAC system. The artificial lighting system consists of compact fluorescence downlights in common areas and 16 mm linear fluorescent ceiling-mount secular lattices in the classrooms and office spaces.

**Figure 7:**
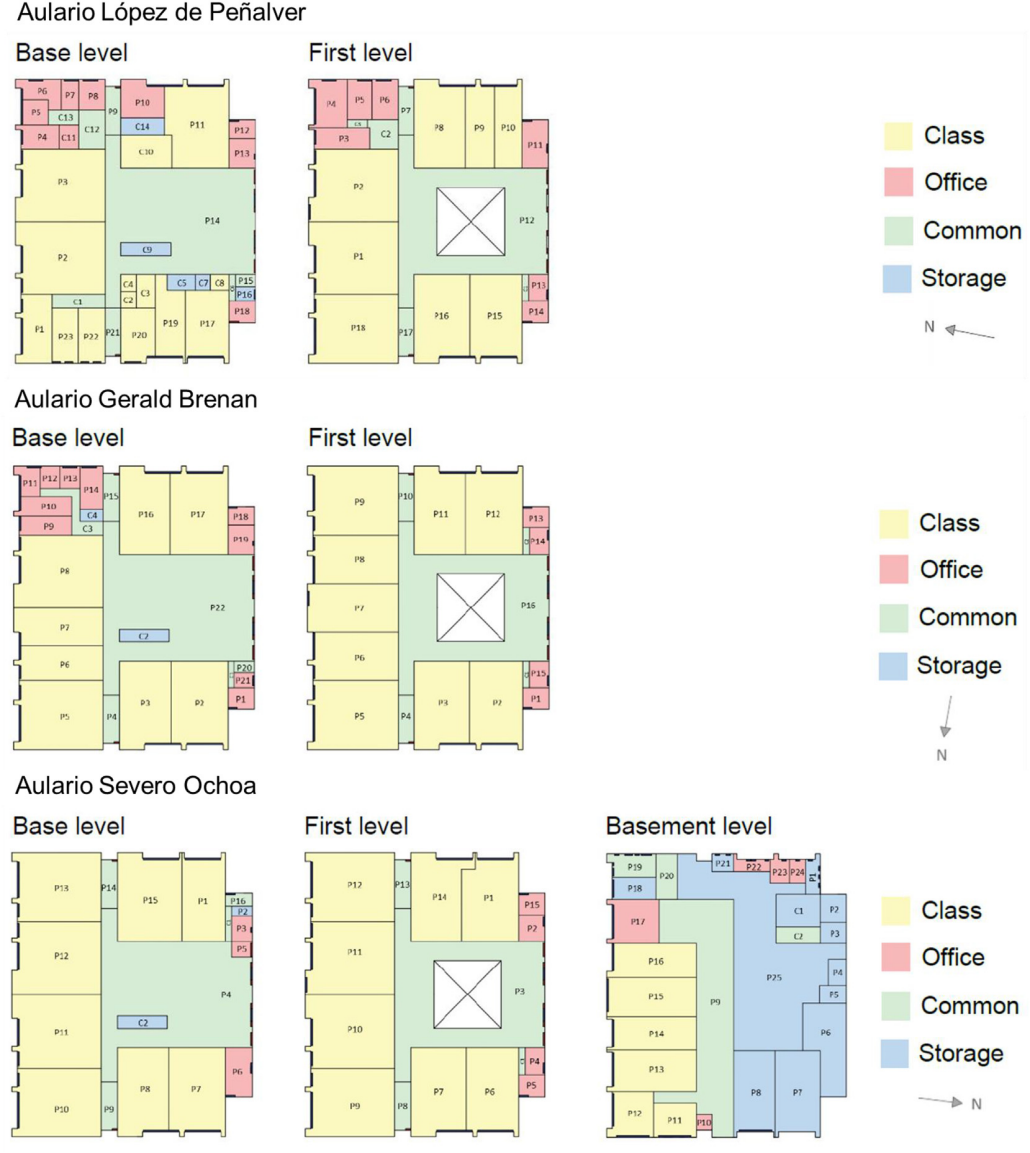
Occupation profile zoning of the studied buildings.

The occupancy profile was set as "Non-Residential Building of Medium Intensity, 16 h" according to the recognized Conditions of Acceptance of Alternative Procedures to LIDER and CALENER (AICIA, 2009). The “one-zone per room” simulation method from Sefaira Systems was chosen to better represent the occupancy profile, considering classroom, office, storage, and common areas, as per Figure 7 and Table 6.

**Table 6:** Occupation profile parameters of the studied buildings.

Occupation profile parameter	Classroom	Office	Common	Storage	Source
Occupant density (m^2^/person)	2.5	10	10	10	ATECYR (2012)
Equipment density (W/m^2^)	4.5	4.5	4.5	4.5	AICIA (2009)
Lighting density (W/m^2^)	15	15	4	15	AENOR (2019)
Ventilation rate (l/s person)	12.5	12.5	12.5	8.3	ATECYR (2012)
Winter heating setpoint (°C)	20	20	20	20	AICIA (2009)
Summer cooling setpoint (°C)	25	25	25	25	AICIA (2009)
Active hours	Labour days: 7 h – 22 h				AICIA (2009)
	Saturdays: 7 h – 14 h				
	Sundays: no occupancy (building closed)				

### 4.2 Current energy consumption

**Figure 8:**
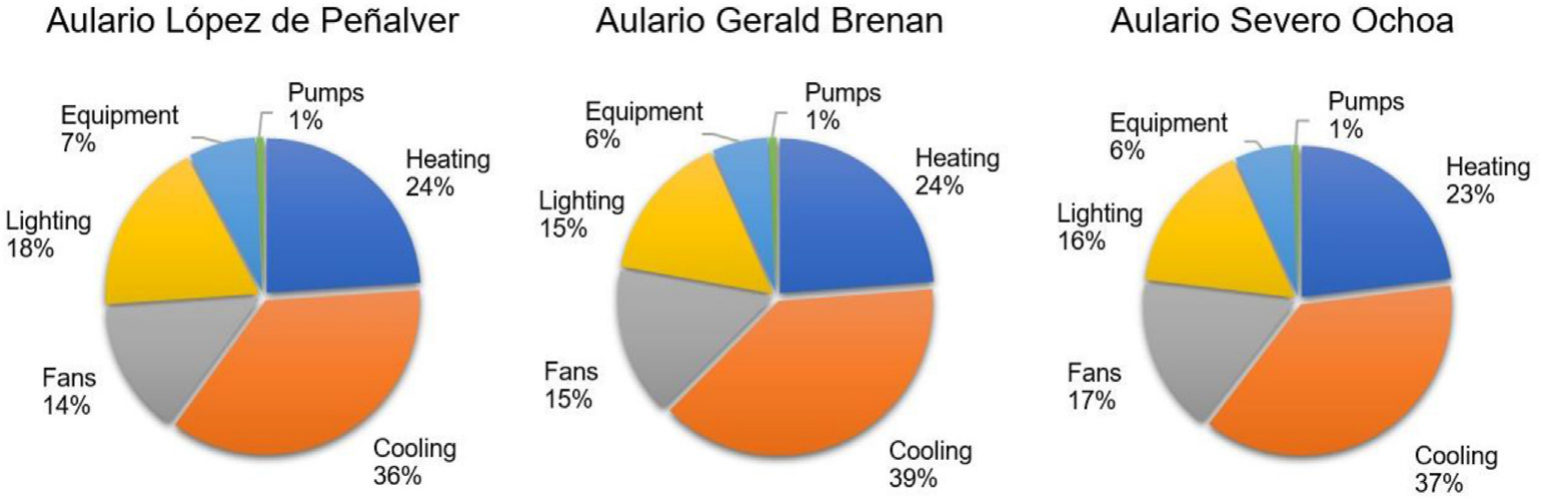
Breakdown of current energy consumption of the studied buildings.

Energy simulation results showed that the buildings' current total primary energy consumption exceeds the nZEB limit of 90 kWh/m^2^ year established by the EPBD. Annually, Aulario López de Peñalver consumes 131 kWh/m^2^, Aulario Gerald Brenan consumes 158 kWh/m^2^, and Aulario Severo Ochoa consumes 141 kWh/m^2^. Approximately 75% of the energy consumption corresponds to cooling, heating, and ventilation. In comparison, the remaining 25% is attributed to lighting and equipment (Figure 8), prioritising the performance increase of the thermal envelope and the HVAC system.

Energy models were validated by comparing the current annual primary energy consumption results for Aulario López de Peñalver against the average annual primary consumption obtained from the building's electricity bills over the past 6 years. The Smart Campus Vice-Rectorate of the University of Málaga maintains annual records of the final energy consumed in kWh by certain groups of buildings on campus, since they are equipped with a collective, rather than individual, electricity metres. The final energy consumed by Aulario López de Peñalver is measured together with the consumption of the Faculty of Medicine and the General Library. No annual consumption data are available for Aulario Gerald Brenan and Aulario Severo Ochoa.

The annual final energy consumption of Aulario López de Peñalver was determined following the extraction procedure proposed by Medrano et al. (2018) to isolate the final energy consumption of individual buildings on Spanish university campuses equipped with collective electricity metres. According to the procedure, collective energy bills were normalized based on the useable area of the buildings (Table 7).

**Table 7:** Annual final energy consumption obtained from the Smart Campus Vice-Rectorate.

Building	Area (m^2^)	Annual Final Energy Consumption (kWh)						
		2013	2014	2015	2016	2017	2018	Average
Faculty of Medicine	20867	1661373	1805106	1566379	1238516	1222592	1245719	1456614
General Library	5906	470219	510900	443333	350538	346030	352577	412266
Aulario López de Peñalver	3195	254377	276384	239832	189632	187194	190735	223026
Total	29968	2385969	2592690	2249545	1778686	1755817	1789031	2091906

Isolation results show that the annual final energy consumption of Aulario López de Peñalver is currently 69.8 kWh/m^2^, equivalent to annual primary energy consumption of 136.4 kWh/m^2^. Compared to the annual primary energy consumption of 131 kWh/m^2^ obtained with the Sefaira software, a 3.96% margin of error is conserved for the models.

### 4.3 Rehabilitation options considered

A total of six nZEB reform options showing their effectiveness in the energy rehabilitation of Mediterranean schools were selected following a thorough review of the literature presented in Table 1. The six proposed options are presented in Table 8 and centre on increasing the thermal envelope and HVAC system's performance and incorporating in situ renewable energy.

**Table 8:** Rehabilitation options considered.

Reform option		Parameter	Current value	New value
P1	Solar PV panels on the roof	Annual production capacity, Cp (kWh/m^2^ of total building area)	0	65
P2	Double-pane argon-cavity low-emissivity windows with PVC frame	Thermal transmittance, U (W/m^2^K)	5.70	1.40
		Solar heat-gain coefficient, g	0.85	0.60
P3	80 mm exterior roof XPS insulation boards	Thermal transmittance, U (W/m^2^K)	0.84	0.34
P4	60 mm exterior façade thermal insulative mortar	Thermal transmittance, U (W/m^2^K)	0.54	0.30
P5	High-efficiency air-air electric heat pump	COP	2.00	4.50
		SCOP	NI	4.44
		EER	1.80	4.15
		SEER	NI	8.29
P6	Building-wide LED lighting system	Power (W/m^2^)	15.00	5.70

NI- No information available.

**Figure 9:**
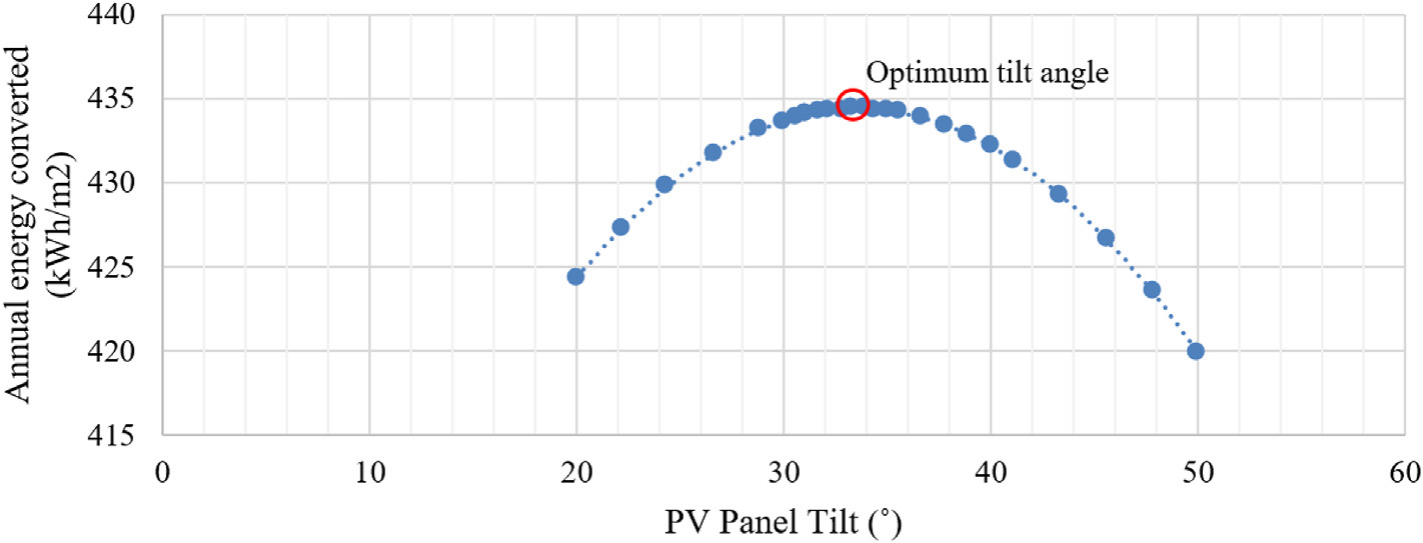
Annual energy converted by unit area of PV panel as a function of panel tilt.

Option P1 consists of installing grid-connected monocrystalline silicon PV panels featuring a rated power of 400 W, a nominal efficiency of 20%, and an annual degradation rate of 0.65%. Following optimization with Sefaira Systems software (Figure 9), the installation setup consists of a due south orientation and a tilt angle of 33.5°. A total of 465 m^2^ of panels will be installed at Aulario López de Peñalver and Gerald Brenan each. In contrast, 725 m^2^ will be installed at Aulario Severo Ochoa to surpass the minimum required annual production capacity of 30 kWh/m^2^ as per Recommendation (EU) 2016/1318, considering the space availability and structural capacity of the roof while avoiding mutual shading.

**Figure 10:**
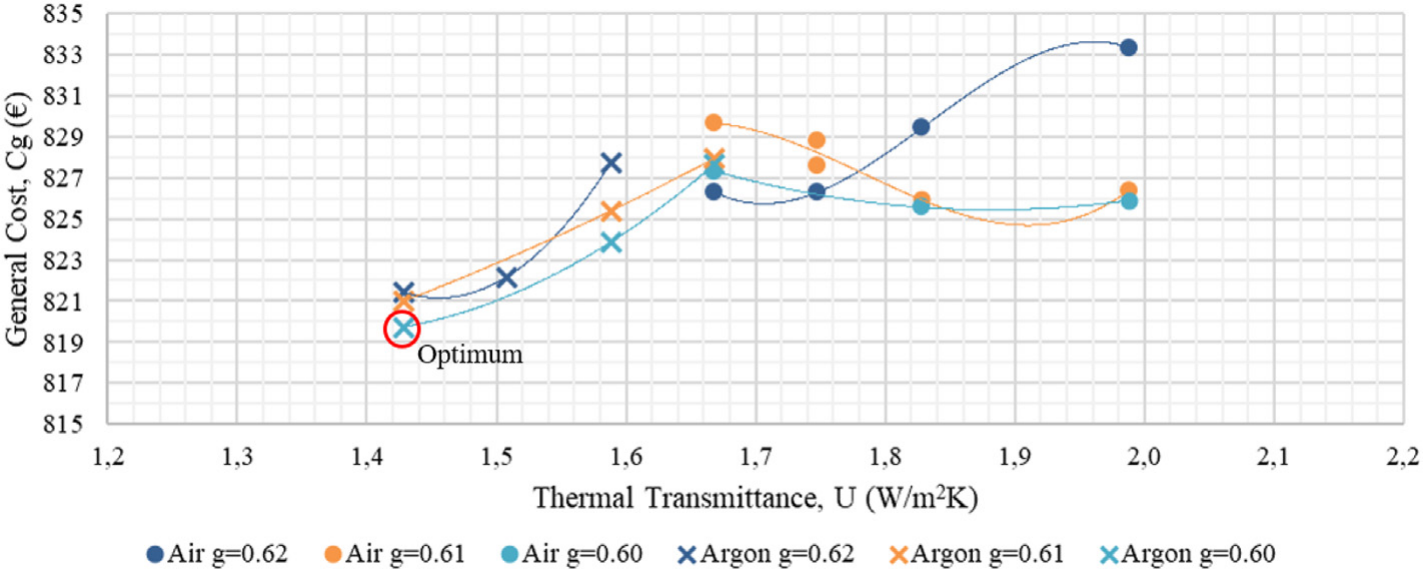
Cost-optimal window thermal insulation.

**Figure 11:**
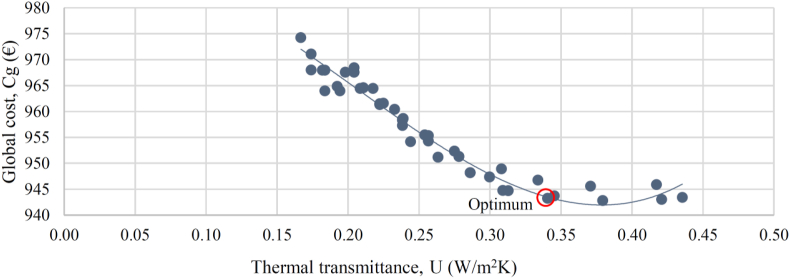
Cost-optimal roof thermal insulation level.

**Figure 12:**
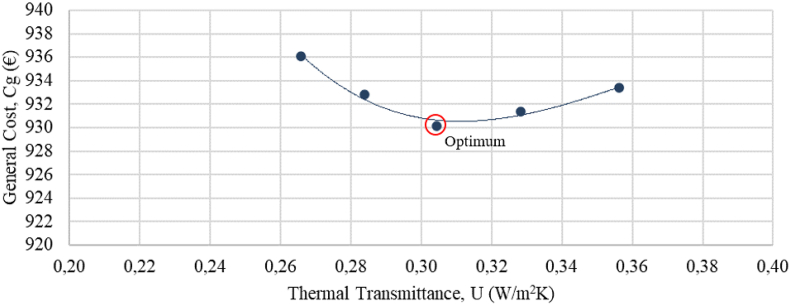
Cost-optimal facade thermal insulation level.

Target thermal transmittance values were optimized through Sefaira Systems software by varying the insulation level of the facades, the roof, and the windows (Figures 10, 11, 12). All windows will be replaced with double-pane argon-cavity (8/14/4) low-emissivity (e < 0.01) windows with PVC frames (P2), 80 mm XPS panels (thermal conductivity of 0.036 W/mK) will be installed on the exterior of the roof (P3). Exterior thermal insulative mortar with a 60 mm thermal mortar finishing (thermal conductivity of 0.046 W/mK) will be applied on facade walls (P4). This thermal insulative mortar system facilitates the application process with a minimal intervention on the existing facades. The current electric air-air heat pump will be replaced with a high-efficiency Model (P5). All downlights in common areas will be equipped with LED bulbs, and *φ*26 mm LED tubes will be installed in all ceiling-mount specular lattices in classrooms and offices (P6).

Reform options P1 through P6 were combined and evaluated in a total of 31 reform combinations, considering two-, three-, four-, five-, and sixfold combinations as per Table 9, always including P1 to guarantee the on-site generation of renewable energy in the energy rehabilitation scenario.

**Table 9:** Rehabilitation combinations evaluated.

Reform combination				
Two-fold	Threefold	Fourfold	Fivefold	Sixfold
P1+P2	P1+P2+P3	P1+P2+P3+P4	P1+P2+P3+P4+P5	P1+P2+P3+P4+P5+P6
P1+P3	P1+P2+P4	P1+P2+P3+P5	P1+P2+P3+P4+P6	
P1+P4	P1+P2+P5	P1+P2+P3+P6	P1+P2+P3+P5+P6	
P1+P5	P1+P2+P6	P1+P2+P4+P5	P1+P2+P4+P5+P6	
P1+P6	P1+P3+P4	P1+P2+P4+P6	P1+P3+P4+P5+P6	
	P1+P3+P5	P1+P2+P5+P6		
	P1+P3+P6	P1+P3+P4+P5		
	P1+P4+P5	P1+P3+P4+P6		
	P1+P4+P6	P1+P3+P5+P6		
	P1+P5+P6	P1+P4+P5+P6		

### 4.4 Economic parameters

Economic parameters utilized for the cost-optimal analysis are presented in Table 10, obtained as per UNE-EN 15459–1:2018 standard and Delegated Regulation (EU) No 244/2012 procedures. Installation costs were obtained from the “Spanish Price Generator” database by CYPE Ingenieros, with construction and rehabilitation cost estimates adjusted for the project location and size (CYPE, 2020).

**Table 10:** Economic parameters implemented in the cost-optimal analysis.

Parameter		Value	Source
Economy	Starting year of renovation, τ0	2020	-
	Building lifespan, τ (years)	30	AENOR (2018)
	Interest rate, r (%)	2	INE (2020)
Energy cost	Electricity price, T (€/kWh of primary energy)	0.1676	Ministerio para la Transición Ecológica y el Reto Demográfico (2018)
	Primary energy cost evolution rate, RXe (%)	2.5	Government of Spain (2020)
	Non-renewable primary energy conversion factor, felct_no_renv	1.954	Ministerio de Industria (2016)
CO_2_ emissions costs	CO_2_ cost 2020–2025, *M* < 2025 (€/tCO_2_)	20	European Commission (2012)
	CO_2_ cost 2025–2030, *M*2025−2030 (€/tCO_2_)	35	
	CO_2_ cost 2030–2050, *M* > 2030 (€/tCO_2_)	50	
	CO_2_ conversion factor, Celct_no_renv (kgCO_2_/kWh final energy)	0.331	Ministerio de Industria (2016)

Proposal	Unit	Installed cost, Ci (€/unit)	Annual maintenance cost, C_m_ (% of C_i_)	Annual replacement cost, C_so_ (% of C_i_)	Annual operation cost, C_f_ (% of C_i_)	Disposal recovery, V_f_ τs (% of C_i_)
P1	kW	1,800	2	-	-	-
P2	m^2^ of window	100–115	0.5	-	-	-
P3	m^2^ of roof	30–55	1.5	-	-	-
P4	m^2^ of facade	63–80	1	-	-	-
P5	total	95,000	2	-	-	-
P6	m^2^ of built area	70	-	10	-	-
Source		CYPE (2020)	AENOR (2018)			

## 5 Results

The objective of this study is to determine the cost-optimal combination of reform options for converting Aulario López de Peñalver, Severo Ochoa, and Gerald Brenan into nearly zero-energy buildings. That is, under Recommendation (EU) 2016/1318, the total post-renovation annual primary energy consumption of these buildings must be reduced to 90 kWh/m^2^ or less, of which the non-renewable share should not surpass 30 kWh/m^2^. A total of 6 reform options were evaluated. The individual impact of each reform option was first evaluated for each building, with the results shown in Table 11. The retrofit of the HVAC system (P5) proves to be the most effective option, reducing the annual primary consumption by 25.4%–27.0%. In contrast, the second most effective option is the installation of high-efficiency windows (P2), with an annual primary energy saving of 8.8%–20.4%. Increasing the amount of roofing insulation (P3) is the least-effective option, providing less than 3.1% energy savings.

**Table 11:** Annual primary energy savings from reform options P1 through P6.

Reform option	Non-renewable annual primary energy consumption (kWh/m^2^)						Standard deviation
	López de Peñalver		Gerald Brenan		Severo Ochoa		
	Value	Reduction	Value	Reduction	Value	Reduction	
P0	131	-	158	-	141	-	-
P1	66	-49.6%	93	-41.1%	76	-46.1%	3.5%
P2	112	-14.7%	126	-20.4%	128	-8.8%	4.8%
P3	127	-3.1%	155	-2.2%	138	-1.9%	0.5%
P4	125	-4.5%	147	-7.2%	134	-4.8%	1.2%
P5	98	-25.4%	116	-27.0%	104	-26.1%	0.6%
P6	117	-11.2%	149	-5.9%	126	-10.5%	2.3%

On the other hand, the PV Panels (P1) can supply 40–50% of the current energy consumption. Electricity consumption from lighting can be decreased by up to 64.8% via LED bulb installation (P6). However, decreased internal thermal gains from LED lights compared to current fluorescent bulbs increase heating demands by 18.0%, hence limiting the overall energy savings from P6 to an average of 9.2%. Table 12 shows the breakdown of total energy consumption for reform options P1 through P6.

**Table 12:** Breakdown of total energy consumption for reform options P1 through P6.

Reform option	Breakdown of total energy consumption		
	López de Peñalver	Gerald Brenan	Severo Ochoa
P0			
P1			
P2			
P3			
P4			
P5			
P6			

With a standard variation of less than 3.5%, all reform options except P2 show a relatively equal impact across all three buildings. The more elevated variability of 4.8% for P2 can be attributed to the difference in the proportion and orientation of windows between each building. The semibasement of the Aulario Severo Ochoa significantly decreases the proportion of windows and their influence on the overall performance of the thermal envelope, corresponding to an inferior energy savings of 8.8% due to window replacement. Given that Aulario Lopez de Peñalver and Gerald Brenan have the same percentage of windows, the lower energy savings observed by Lopez de Peñalver (14.7%) may be because the facade with the most glazing (facade C) is oriented north, with no direct sunlight exposure compared to that of Gerald Brenan, which has an eastern orientation and a corresponding energy savings of 20.4%. As such, the findings indicate that window replacement may provide the greatest energy savings for academic buildings with a higher total glazing percentage under direct sun exposure.

**Figure 13:**
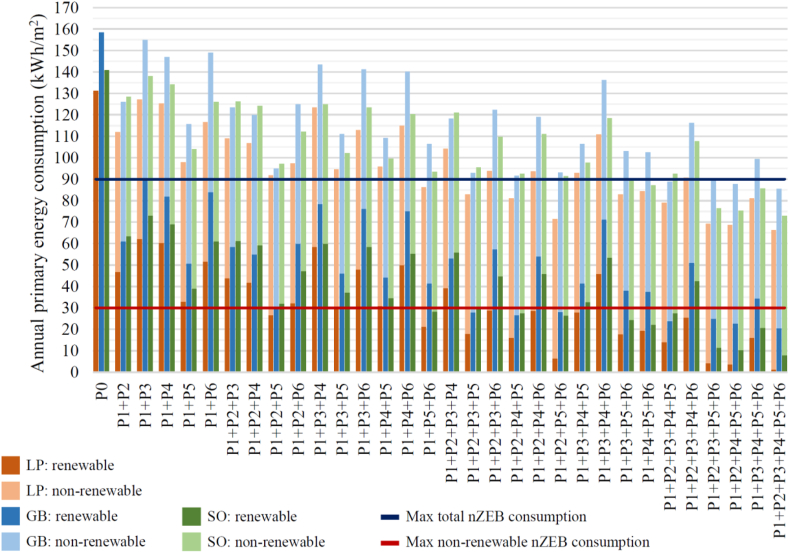
Pre- and post-rehabilitation annual primary energy consumption of Aulario López de Peñalver (LP), Gerald Brenan (GB) and Severo Ochoa (SO) compared to nZEB thresholds.

Figure 13 presents the annual primary energy consumption of Aulario López de Peñalver, Gerald Brenan, and Severo Ochoa after applying the 31 reform combinations to identify those that meet nZEB requirements. With the lowest initial primary energy consumption, Aulario Lopez de Peñalver requires the lowest level of intervention with 11 possible combinations of at least 3 reform options each, providing a total energy savings of 84.0%–99.2%. Aulario Severo Ochoa has the highest initial energy demand, requiring the highest level of intervention with at least 5 reform options to be implemented in 4 possible combinations, achieving a total energy savings of 84.2%–86.7%. In the case of Aulario Severo Ochoa, a mid-level intervention intensity is required, comprised of at least 4 reform options in 6 possible combinations to achieve energy savings of 83.0%–94.3%. The non-renewable primary energy consumption threshold of 30 kWh/m^2^ cannot be attained without installing PV panels (P1) for all studied retrofit scenarios. Only three retrofit scenarios simultaneously meet the nZEB requirements for all three buildings: P1+P2+P3+P4+P5+P6, P1+P2+P4+P5+P6, and P1+P2+P3+P5+P6.

**Figure 14:**
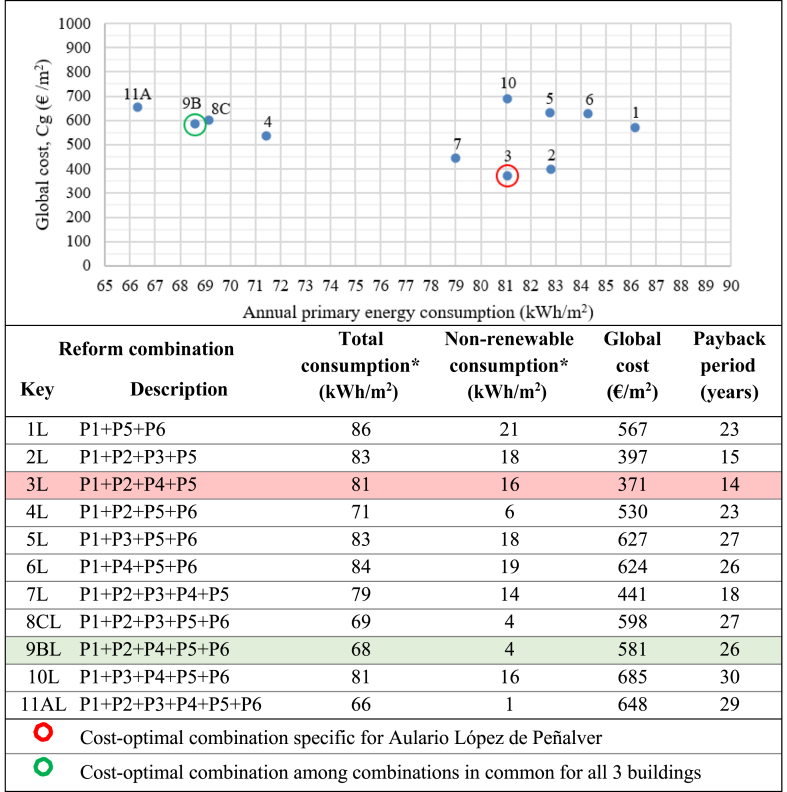
Cost-optimization results for Aulario López de Peñalver. ∗Annual primary energy.

**Figure 15:**
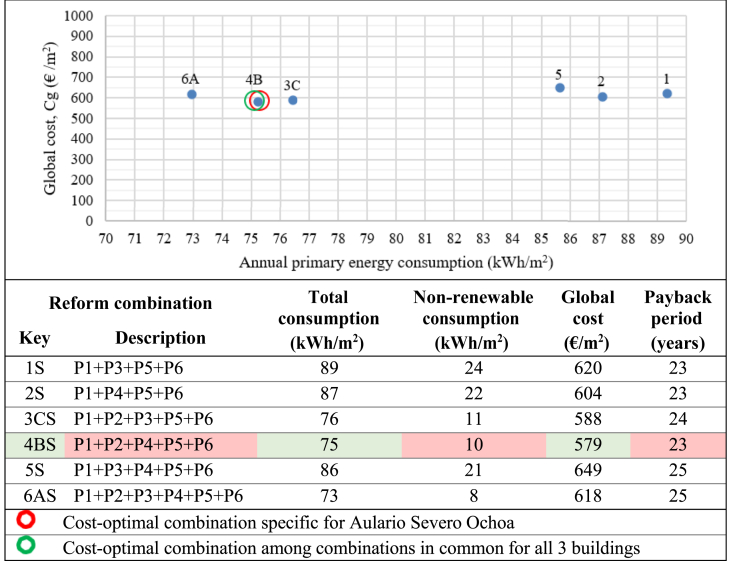
Cost-optimization results for Aulario Gerald Brenan. ∗Annual primary energy.

**Figure 16:**
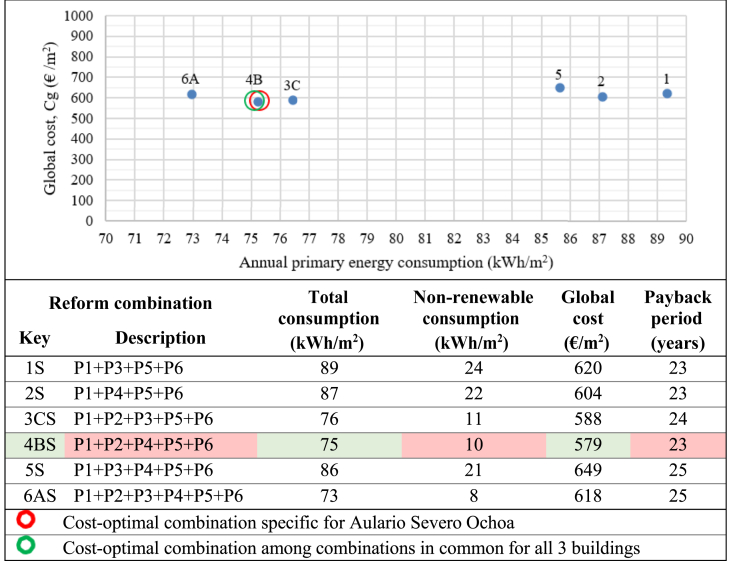
Cost-optimization results for Aulario Severo Ochoa. ∗Annual primary energy.

Cost-optimization was then performed on the 11 possible combinations meeting the nZEB requirement for Aulario Lopez de Peñalver, the 4 possible combinations for Aulario Severo Ochoa, and the 6 possible combinations for Gerald Brenan. The results of the cost-optimization analysis for the reform combinations meeting nZEB requirements are presented in Figures 14, 15 and 16. The cost-optimal combination for each building is marked in red, corresponding to the lowest point in the data plot as per methodological framework established in Commission Delegated Regulation (EU) No 244/2012 and illustrated in Figure 1 above. As previously mentioned, only three retrofit scenarios simultaneously meet the nZEB requirements for all three buildings, namely combinations A (P1+P2+P3+P4+P5+P6), B (P1+P2+P4+P5+P6), and C (P1+P2+P3+P5+P6). The cost-optimal strategy among these three common combinations has been identified and marked in green with the aim of identifying the cost-optimal strategy that would suit all three buildings simultaneously.

Compared to the current state, the proposed nZEB reforms could reduce electricity bill costs by 87.7%–92.7%. The optimal combination for Aulario Lopez de Peñalver is P1+P2+P4+P5 with 87.8% energy savings, a global cost of 371 €/m^2^, and a payback period of 14 years. The optimal combination for Aulario Gerald Brenan is P1+P2+P3+P4+P5 with an associated energy savings of 84.8%, the global cost of 506 €/m^2^, and a payback period of 15 years. The optimal reform combination for the Aulario Severo Ochoa is P1+P2+P4+P5+P6, with 92.9% energy savings at a global cost of 579 €/m^2^ and a 23-year payback period. It can be noted that the cost-optimal nZEB performance does not coincide with the maximum achievable energy efficiency. For example, although it is technically feasible to reduce the energy consumption of the Aulario López de Peñalver to only 1 kWh/m^2^, it is not economically profitable due to the incremental cost of renovation. Therefore, the optimum level implies consumption of more than 16 kWh/m^2^.

Each optimal combination stems from the same base (P1+P2+P4+P5), corresponding to the reform options with the highest individual performance. Soto Francés et al. (2020) argue that the same retrofit strategy applied to different academic buildings of similar typology in similar climatic conditions results in different energy savings. This conclusion agrees with the results obtained in the present study, which finds that the cost-optimal reform combination is specific and particular to each building. The difference in energy performance and optimal nZEB reform strategy could be primarily due to the different orientations of the almost identical buildings and second due to the small constructive variations between them.

Results may suggest that the most favourable orientation is to avoid locating classrooms and offices to the south of the building to minimize cooling loads from solar gains in the hot Mediterranean summers (Table 13). North-oriented classrooms and offices feature the lowest current energy consumption, while the cost-optimal nZEB strategy features the lowest global cost and payback period. Both Aulario Gerald Brenan and Aulario Severo Ochoa feature classrooms and offices located to the south of the building, having the highest current energy consumption of 158 and 141 kWh/m^2^, respectively. The cost-optimal nZEB retrofits for two buildings require the highest global cost at 506 and 579 €/m^2^, respectively, as well as the highest payback periods of 15 and 23 years, respectively. On the other hand, Aulario López de Peñalver features all classrooms and offices located towards the North of the building, having the lowest current energy consumption of 131 kWh/m^2^. The cost-optimal nZEB strategy for Aulario López de Peñalver consequently features the lowest global cost and payback period, at 371 kWh/m^2^ and 14 years, respectively.

**Table 13:** Impact of building orientation on energy performance and optimal nZEB reform strategy.

Building	López de Peñalver	Gerald Brenan	Severo Ochoa

Current total annual primary energy consumption (kWh/m^2^)	131	158	141
Current non-renewable primary energy consumption (kWh/m^2^)	131	158	141
Reformed total annual primary energy consumption (kWh/m^2^)	81	89	75
Reformed non-renewable primary energy consumption (kWh/m^2^)	16	24	10
Number of reform options	4	5	5
Optimal reform combination	P1+P2+P4+P5	P1+P2+P3+P4+P5	P1+P2+P4+P5+P6
Global cost (€/m^2^)	371	506	579
Payback period (years)	14	15	23
Electricity bill savings (%)	88	85	93

Soto Francés et al. (2020) argue that nZEB renovations in academic buildings are not economically feasible unless PV modules are installed for on-site renewable energy provision. These findings are consistent with the results obtained in the present study, which show that rooftop PV panel installation is indispensable for successful nZEB retrofitting to meet the maximum nZEB primary non-renewable energy consumption limits. Considering that the renewable energy production capacity is limited by the available roof area and maximum PV technology efficiency, the optimal nZEB renovation strategy focuses on minimizing the energy demand of the building. This is best achieved by improving the energy performance of the thermal envelope and HVAC system, given that almost 75% of the current energy consumption corresponds to heating, cooling, and ventilation.

High payback periods are expected among all combinations meeting nZEB requirements for all three buildings, ranging from 14 up to 30 years. In other words, the investment costs for the identified nZEB reformations would not be recovered until half of the buildings' expected lifetime under the best-case scenario and until the buildings' expected lifetime under the worst-case scenario. It is worth noting that the cost-optimal combinations correspond to the lowest payback period for each building.

**Figure 17:**
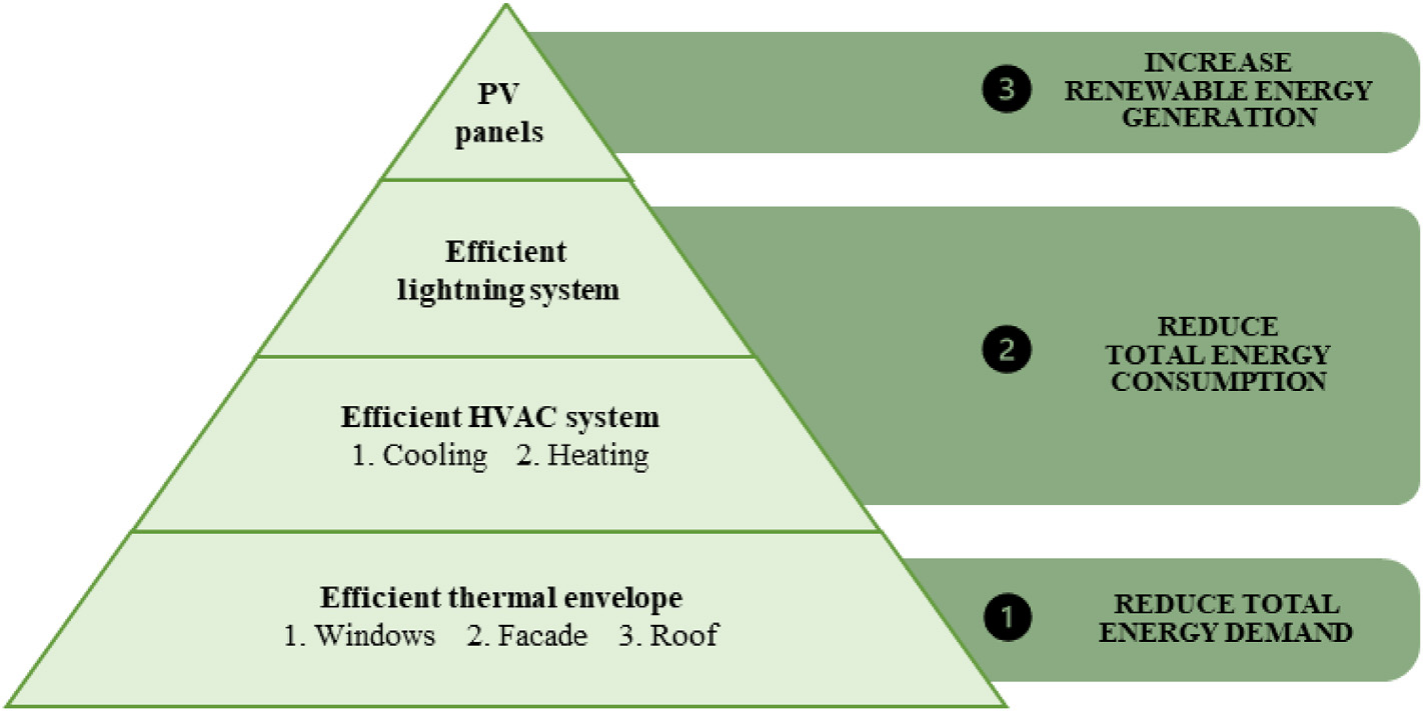
General nZEB retrofitting strategy for university buildings in the Mediterranean climate.

A general strategy for the energy rehabilitation of university academic buildings in Mediterranean climate zones is proposed in Figure 17.

## 6 Discussion

It is possible to meet and surpass the nZEB requirements set by the European Commission by implementing the proposed cost-optimal retrofitting strategies identified for each lecture hall due to the present study. The annual non-renewable primary energy consumption of Aularios López de Peñalver, Gerald Brenan and Severo Ochoa would be reduced by 88%, 85% and 93% to values of 16 kWh/m^2^, 24 kWh/m^2^ and 10 kWh/m^2^, respectively. These results are consistent with the nZEB consumption values determined in the benchmark studies, as the implementation of the optimal reform combinations identified in the present study can reduce the annual non-renewable primary energy consumption of academic buildings located in Mediterranean Europe by 63–91% to a value between 12 and 29 kWh/m^2^ (Table 14). Ascione et al. (2017) report similar results for the retrofitting of a university building in Benevento, Italy, highlighting that the two most cost-effective options consist of upgrading the HVAC system and installing photovoltaic panels on the roof.

**Table 14:** Technical feasibility of nZEB reform strategies for academic buildings situated in Mediterranean Europe.

Case study	Initial annual primary energy consumption (kWh/m^2^)		Post-renovation annual primary energy consumption (kWh/m^2^)		Energy savings (%)	
	Total	Non-renewable	Total	Non-renewable	Total	Non-renewable
López de Peñalver	131	131	81	16	-38%	-88%
Gerald Brenan	158	158	89	24	-44%	-85%
Severo Ochoa	141	141	75	10	-47%	-93%
Berardi et al. (2017)	146	146	78	20	-47%	-86%
Gaitani et al. (2015)	128	128	52	21	-59%	-84%
Ascione et al. (2017)	138	138	No data	12	N.d.	-91%
Rospi et al. (2017)	110	110	25	25	-77%	-77%
Congedo et al. (2016)	418	418	63	No data	-85%	No data
ZEMedS (2015a)	76	76	66	28	-13%	-63%
ZEMedS (2015b)	183	183	69	29	-62%	-84%
ZEMedS (2015c)	130	130	68	28	-48%	-78%

In terms of economic feasibility, the overall cost of the proposed nZEB renovations is within literature ranges, and a slightly better than average payback period is estimated (Table 15). Compared to the current state, the proposed cost-optimal nZEB renovations could reduce classroom electricity bills by 87.7%–92.7%, whereby a payback period between 14 and 23 years is expected. While it is technically feasible to meet nZEB requirements, the economic sustainability is questionable, as payback times exceed half the lifetime of the buildings. This calls for the future exploration of ways to diminish initial investment costs and augment the economic rentability of Europe's nZEB ambition.

**Table 15:** Economic feasibility and optimal energy efficiency of the thermal envelope.

Case study	Global cost (€/m^2^)	Payback (years)	Optimal thermal transmittance, U (W/m^2^K)		
			Roof	Facade walls	Windows
Aulario López de Peñalver	371	14	0.34	0.30	1.4
Aulario Gerald Brenan	506	15	0.34	0.30	1.4
Aulario Severo Ochoa	579	23	0.34	0.30	1.4
Berardi et al. (2017)	No data	41	No data	0.20	1.6
Gaitani et al. (2015)	879	27	0.22	0.30	1.5
Ascione et al. (2017)	288	No data	0.27	0.30	1.7
Rospi et al. (2017)	No data	No data	No data	No data	No data
Congedo et al. (2016)	398	No data	No data	0.33	1.3
ZEMedS (2015a)	264	36	0.22	0.30	1.5
ZEMedS (2015b)	528	23	0.22	0.30	1.5
ZEMedS (2015c)	582	31	0.22	0.30	1.5

Developed optimisation curves for the roof, facades, and windows (Figures 10, 11, and 12) reveal that when carrying out a nZEB renovation, it is important to detect the cost-optimal insulation level, given that the energy performance of a building can be sharply increased up to the optimal level, after which the incremental investment costs are not compensated by decreasing the energy benefits of added thermal insulation.

Table 15 shows the optimal thermal transmittance for the roof, facade walls, and windows of academic buildings situated in the Mediterranean climate. The optimal transmittance for the facades of Aularios López de Peñalver, Gerald Brenan and Severo Ochoa was determined at 0.30 W/m^2^K, coinciding exactly with the value reported in Ascione et al. (2017), Gaitani et al. (2015) and ZEMedS (2015a, 2015b, 2015c). The optimal transmittance for the windows was determined at 1.4 W/m^2^K, falling within the optimal range of 1.3–1.7 W/m^2^K reported in the benchmark studies, a range attributed to the wide diversity of window models and prices available on the market. Nevertheless, all benchmark studies recommend the installation of double-glazed windows with 14–16 mm argon chambers and PVC or aluminium frames with thermal breaks. For the roof, the optimum transmittance for three lecture halls was determined at 0.34 W/m^2^K, being 0.12 W/m^2^K higher than the optimum levels from the benchmark studies, which could be attributed to the different types of roofing insulation considered. This study considers XPS panels of higher investment cost than the expanded polyethylene panels (EPS) considered by ZEMedS (2015a, 2015b, 2015c). Starting from a higher investment cost, the cost-efficiency optimisation curve shifts in favour of higher transmittance levels. Ascione et al. (2017) also proposed XPS panels and reported an optimal transmittance level of 0.27 W/m^2^K, which is close to the value obtained in this study.

Several measures were adopted to minimise variability and uncertainty in the results. The energy models and the simulation procedure with Sefaira software were validated against the historic energy electricity bills available, which with an error inferior to 4%, and the energy model results were considered reliable. It should be noted that the cost optimisation of the reform proposals is based on budgetary assumptions that are subject to change according to economic fluctuations. By adopting the procedural framework set out in Delegated Regulation (EU) No244/2012 and the UNE-EN 15459–1:2018 standard, the uncertainty of the economic assessment is reduced. However, changes in the energy price, discount rate, and technology prices considered over the lifetime of the lecture halls can significantly shift the cost-optimization curves.

Sefaira software only partially simulates the influence of the surrounding vegetation, as the shading pattern is considered, but evapotranspirative cooling, which could significantly reduce the cooling demand, is not considered. Due to the limitations of the simulation engine, the theoretical results provided by Sefaira software do not consider the interaction between the user and the building. Changes in user behaviour can provide significant energy savings at almost zero investment cost, according to Ascione et al. (2015). For example. Pisello et al. (2016) reveal that there is up to a 300% variability in individual perception of indoor microclimate, personal preferences for thermal comfort, individual preferences for lighting levels and habits of switching on/off lamps, opening/closing doors and opening/closing windows among users of a university building in Perugia, Italy. Becchio et al. (2016) argue that user behaviour is critical to the success of a nZEB building but is often labelled "the missing piece in the sustainability puzzle" due to the complexity of addressing human behaviour.

## 7 Conclusions

This study presents a reference case to close the gap between the low energy performance of Mediterranean Spanish university campuses and the nZEB goal established by the EPBD. Energy rehabilitation strategies were optimized through simulations with Sefaira Systems software for Aularios López de Peñalver, Gerald Brenan and Severo Ochoa of the University of Málaga, considering options that do not conduct any alterations to the architectural form.

The results indicate that it is technically feasible to reach the nZEB goal in Mediterranean academic buildings. Although a general strategy for nZEB renovations is identified, it is concluded that the cost-optimal strategy is unique to the specific conditions of each building. It is shown that PV panels are indispensable and capable of meeting 40–50% of the building's current energy consumption. However, the most cost-effective strategy emphasizes reducing the energy demand instead of increasing renewable energy generation. Considering that 75% of the primary energy use profile of the buildings corresponds to heating, cooling and ventilation demands, the top two reform options identified are:

1. Replacing the HVAC system with a high-efficiency electric heat pump for an energy savings of 25.4%–27.0%
2. Upgrading all windows with double-pane, low-emissivity models with PVC framing for an energy savings of 8.8%–20.4%.

The cost-optimal thermal transmittance values for Aularios López de Peñalver, Severo Ochoa, and Gerald Brenan were determined to be 0.30 W/m^2^K for facades, 0.34 W/m^2^K for roofing, and 1.4 W/m^2^K for windows. Despite having an identical floorplan and profile use, the cost-optimal reform strategy was found to be specific to each building, whereby building orientation proved to be a governing factor in the buildings' energy performance, in combination with slight nuances in actual use and distribution.

The cost-optimal strategy for Aulario López de Peñalver consist of installing solar PV panels on the roof, replacing existing windows double-pane argon-cavity low-emissivity windows with PVC frame, adding external thermal insulative mortar on the facades, and upgrading the HVC system to a high-efficiency air-air electric heat pump. The cost-optimal strategy for Aulario Gerald Brenan additionally requires exterior roofing insulation boards to reduce the additional heat gains from its unfavourable orientation. Aulario Severo Ochoa additionally requires building-wide LED lighting systems, given its increased lighting loads arising from its semi-basement.

The results could suggest that the most favourable orientation is that which locates classrooms and offices away from south-oriented facades to minimize cooling loads in summer, resulting in lower global costs and payback periods for nZEB retrofits.

## Declarations

### Author contribution statement

Daniela Carolina Da Costa Duarte: Conceived and designed the experiments; Performed the experiments; Analyzed and interpreted the data; Wrote the paper.

Carlos Rosa-Jiménez: Analyzed and interpreted the data; Contributed reagents, materials, analysis tools or data; Wrote the paper.

### Funding statement

This work was supported by 1^st^ Smart-Campus Plan of University of Malaga [project Microsol].

### Data availability statement

Data included in article/supplementary material/referenced in article.

### Declaration of interests statement

The authors declare no conflict of interest.

### Additional information

No additional information is available for this paper.
